# Assessing SNP-markers to study population mixing and ecological adaptation in Baltic cod

**DOI:** 10.1371/journal.pone.0218127

**Published:** 2019-06-20

**Authors:** Peggy Weist, Franziska M. Schade, Malte Damerau, Julia M. I. Barth, Jan Dierking, Carl André, Christoph Petereit, Thorsten Reusch, Sissel Jentoft, Reinhold Hanel, Uwe Krumme

**Affiliations:** 1 Thünen-Institute of Fisheries Ecology, Bremerhaven, Germany; 2 Thünen-Institute of Baltic Sea Fisheries, Rostock, Germany; 3 Zoological Institute, University of Basel, Basel, Switzerland; 4 Centre for Ecological and Evolutionary Synthesis, Department of Biosciences, University of Oslo, Oslo, Norway; 5 GEOMAR Helmholtz Centre for Ocean Research, Kiel, Germany; 6 Department of Marine Sciences-Tjärnö, University of Gothenburg, Strömstad, Sweden; Natural History Museum of London, UNITED KINGDOM

## Abstract

Atlantic cod (*Gadus morhua*) is a species of great ecological and economical importance in the Baltic Sea. Here, two genetically differentiated stocks, the western and the eastern Baltic cod, display substantial mechanical mixing, hampering our understanding of cod ecology and impeding stock assessments and management. Based on whole-genome re-sequencing data from reference samples obtained from the study area, we designed two different panels of *Single Nucleotide Polymorphisms* markers (SNPs), which take into account the exceptional genome architecture of cod. A minimum panel of 20 diagnostic SNPs and an extended panel (20 diagnostic and 18 biologically informative SNPs, 38 in total) were developed and validated to distinguish unambiguously between the western and the eastern Baltic cod stocks and to enable studies of local adaptation to the specific environment in the Baltic Sea, respectively. We tested both panels on cod sampled from the southern Baltic Sea (n = 603) caught in 2015 and 2016. Genotyping results showed that catches from the mixing zone in the Arkona Sea, were composed of similar proportions of individuals of the western and the eastern stock. Catches from adjacent areas to the east, the Bornholm Basin and Gdańsk Deep, were exclusively composed of eastern Baltic cod, whereas catches from adjacent western areas (Belt Sea and Öresund) were composed of western Baltic cod. Interestingly, the two Baltic cod stocks showed strong genetic differences at loci associated with life-history trait candidate genes, highlighting the species’ potential for ecological adaptation even at small geographical scales. The minimum and the extended panel of SNP markers presented in this study provide powerful tools for future applications in research and fisheries management to further illuminate the mixing dynamics of cod in the Baltic Sea and to better understand Baltic cod ecology.

## Introduction

In fisheries, management units are often not equivalent to biological populations or stock units. This is of particular concern when stocks mix and mixed catches hamper accurate stock assessments and sustainable fisheries management [1]. Understanding the internal dynamics of individual stocks, which often differ in reproductive and growth parameters, is essential to prevent the overexploitation of cryptic populations and thus, the depletion of genetic resources [2]. The loss of stock components can be detrimental by negatively affecting the recruitment potential and eventually reducing the genetic diversity of populations. To resolve the composition of mixed-stock fisheries and to identify the different source populations, molecular methods have been proven informative [3].

Multilocus assignment tests are widely used to identify the genetic origin of an individual. In populations with pronounced genetic structure, a relatively low number of polymorphic markers is sufficient to determine the origin, whereas in more weakly structured populations the number of markers required to assign unknown samples with confidence increases remarkably fast [4]. Although widely applied, traditional microsatellite analysis may lead to putatively false negatives in the identification of population structure due to methodological pitfalls, such as spuriously increased sample homozygosity caused by the presence of null alleles [5], or an overestimation of the accuracy of the markers [6]. Both effects hinder the correct genetic assignment of individuals.

Modern genetic traceability methods for marine fish apply genome-wide approaches to identify small panels of SNPs with high assignment power [7], representing a promising tool to design custom marker panels to address specific assignment questions. Such techniques facilitate the identification of populations and the assessment of population contributions to mixed-stock fisheries.

**Fig 1:**
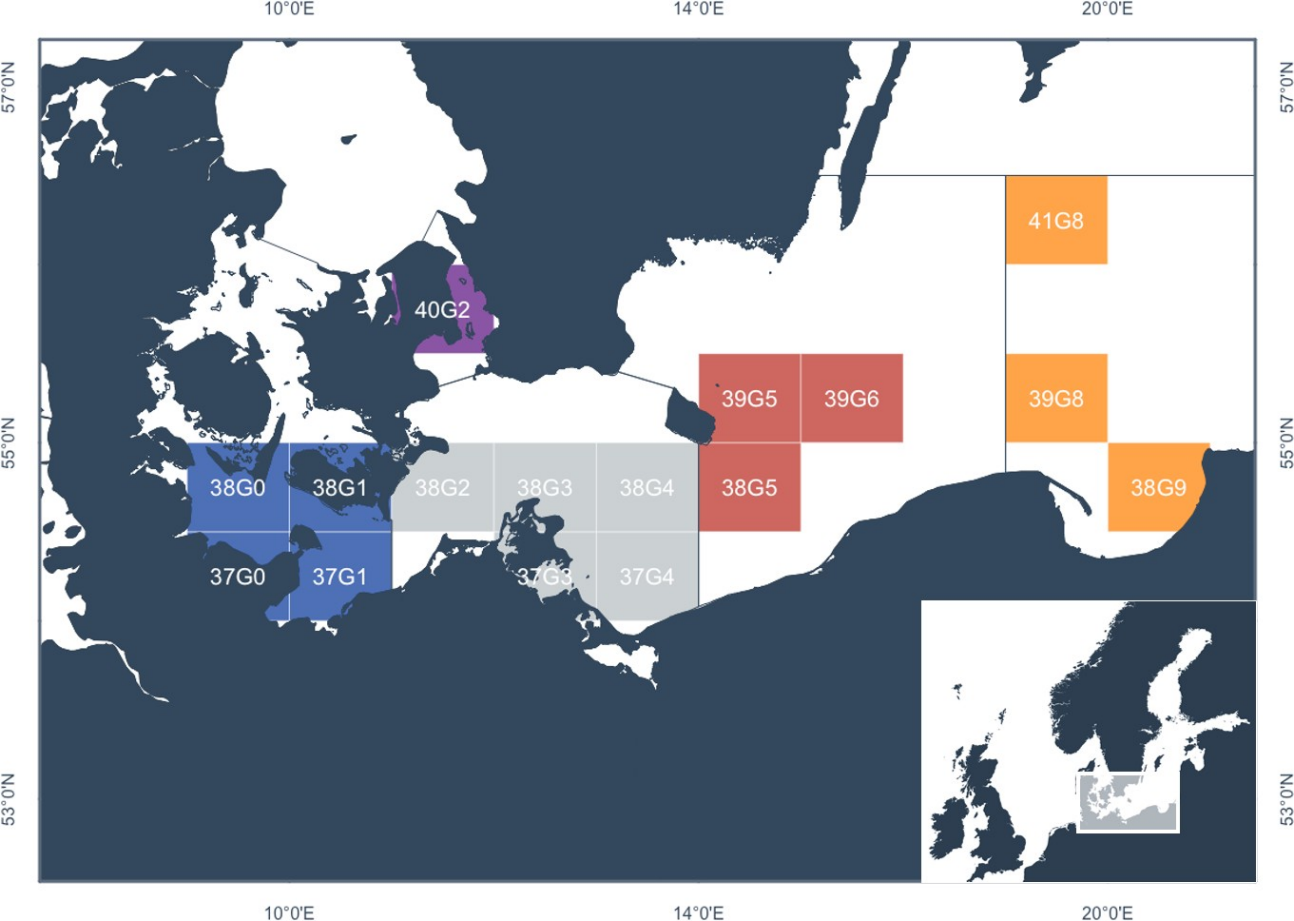
Distribution of sampling locations of genotyped individuals within the Southern Baltic Sea. ICES rectangles in subdivisions 22 (blue), 23 (violet), 24 (grey), 25 (red) and 26 (orange). For details see Table 1.

Atlantic cod (*Gadus morhua*) is a commercially important fish species throughout the North Atlantic Ocean and adjacent waters, including the brackish waters of the Baltic Sea. Baltic cod have developed specific physiological and life-history traits to cope with the unique environmental challenges of the Baltic Sea, such as hypoxic and hypo-osmotic conditions [8,9]. In the Baltic Sea, cod populations are managed as two stocks, which can be categorized by their spatial distribution into a western (ICES subdivisions (SD) 22–24), and eastern stock (SD 24–32, [10]). Whereas the western Baltic cod population mainly uses the waters of the Belt Sea (SD 22) and Öresund (SD 23) for spawning, the eastern Baltic cod population uses the deeper basins in SD 25 and further east for spawning (see Fig 1; [11]). The stocks differ in stock size and life-history patterns, such as growth and maturation patterns [12,13]. The Arkona Sea (SD 24) is located between the western SD 22 and the eastern SD 25, which makes it a potential mixing area. Efforts to estimate mixing proportions of the two stocks have confirmed that the Arkona Basin is indeed used by both western and eastern Baltic cod, but spatio-temporal mixing dynamics are still unclear [14]. Thus, there is an apparent need for cost-effective and accurate methods to assign individuals inhabiting this area to the western or the eastern Baltic cod stock, as well as to determine the gene flow between the two stocks, to improve our knowledge of the population ecology, determine the stock contributions to catches from this area and to consider the results in the fisheries management of the two Baltic cod stocks [15].

Previous molecular approaches for discriminating between western and eastern Baltic cod made use of various marker types. While allozyme markers failed to reveal genetic differentiation among populations [16], the application of highly polymorphic microsatellite loci successfully pointed at a genetic structure of distinct cod populations. By genotyping individuals at nine neutral microsatellite markers, the degree of differentiation between cod from the Belt Sea and the Bornholm Basin was estimated at 3% and suggested the existence of a hybrid zone located in the western Baltic between North Sea cod and Baltic cod [17]. Reduced genome analyses based on a 10K SNP-array of randomly distributed markers throughout the genome revealed a high degree of genetic differentiation of the two Baltic cod populations and allowed an unambiguous assignment of 70% of the individuals to their population of origin [18]. Analyses based on genotype data generated with a high-graded SNP-panel designed to differentiate between individuals from the western and the eastern Baltic cod stock, provided further insights into the dynamics within the mixing zone [14,19]. However, the genomic resources for Atlantic cod have been substantially improved since the original generation of the markers used for the differentiation of Baltic cod populations [7,19,20], and have revealed a complex genome architecture [21–23]. Taking such genomic features into account when designing diagnostic SNP-based assays is important to improve the accuracy and power of individual assignment results compared to existing SNP-assays [14,19].

Here, we present the so far smallest existing set of highly diagnostic SNP markers based on low coverage whole-genome re-sequencing data, to unambiguously distinguish between individual western and eastern Baltic cod. We tested the usefulness of this new minimum panel to assign individuals to their respective population of origin by genotyping cod individuals comprehensively sampled from the southern Baltic over two successive years with specific focus on samples collected in the mixing area of the two Baltic cod populations, the Arkona Sea. It enables the discrimination of the two Baltic cod stocks at a high confidence level and with minimum effort. In addition, we tested the efficiency of an extended panel that also includes biologically informative markers. These included sex discriminating SNPs [24], SNPs located within three identified chromosomal inversions [23,25] as well as candidate genes suggested to be of importance for local adaptation to the divergent pressures along the environmental gradient of the Baltic Sea [9].

## Materials and methods

### Sampling and genotyping of Baltic cod

We genotyped 603 cod individuals from the southern Baltic Sea (ICES SD 22–26, Table 1 and Fig 1) obtained from survey, commercial and recreational catches in 2015 and 2016 with the minimum diagnostic panel of 23 SNPs and an extended marker set of 48 SNPs (see S1 File and S1–S4 Tables for details on whole-genome re-sequencing of cod samples, variant calling, SNP identification and power analysis of the selected SNPs). Spawning individuals (maturity stage 5 = pre-spawning and 6 = spawning [26]) were sampled in SD 22 and 25, assuming that reference specimens for the western and eastern Baltic cod stock were caught during their respective spawning season. In the Arkona Basin (SD 24), cod were collected during the whole year from active and passive catches and from individuals with various maturity stages. Pieces from gill or muscle tissue were stored in pure ethanol at -20°C until processing. DNA was isolated using the Invisorb Spin DNA Extraction Kit (Stratec Molecular, Germany) according to the manufacturer’s instructions. For genotyping cod with the 48-SNP panel, custom allele-specific SNPtype assays were designed for use on a Fluidigm BioMark HD System (S5 Table). Prior to genotyping, specific target amplifications were run to optimize DNA-concentrations for each assay. We used 2.86 μL of diluted PCR products (1:100) and prepared 96.96 IFC dynamic arrays with 96 samples against a 48-SNP panel in accordance with the manufacturer’ protocol and applied the suggested PCR conditions. Genotypes were first auto-called by SNP GENOTYPING ANALYSIS (Fluidigm) with a confidence threshold set at 95. All auto-calls were checked manually and overall data quality, locus and sample performances were evaluated. In total, ten loci were excluded from downstream-analyses (three diagnostic SNPs, seven biologically informative SNPs): all sex loci, monomorphic loci and loci with more than 50% of missing genotype data (see S5 Table). SNPs that did not cluster well were also removed from the dataset. Low-performing individuals with < 50% genotype data were removed. Thus, three out of 23 diagnostic SNPs were excluded from the panel. For convenience, the panel comprising only the resulting diagnostic SNP panel is hereafter referred to as minimum panel which contains 20 SNPs. The panel comprising the full chip, i.e. diagnostic SNPs, plus SNPs in inversions and candidate genes, is referred to as extended panel (38 SNPs).

**Table 1:** Summary of samples used for genotyping (N = 603). ICES subdivision, approximate water depth, ICES rectangle, sampling location and number of sampled individuals (N) are given together with sampling year and month, length information and standard deviation (SD). The proportion of spawning individuals was calculated from individual maturity stages, which were categorized based on their gonadal state of maturation (maturity stages V and IV, following [26]).

Subdivision	Water depth [m]	Rectangle	Longitude	Latitude	Year	Month	N	Range length [cm]	Mean length ± SD [cm]	Males [%]	Spawning [%]	Fishing gear	Sample type
22	20–25	37G0	54.416	11.429	2016	Mar	12	31–79	65.05 ± 11.89	75	100	Bottom trawl	Survey
		37G1	54.12	11.215	2016	Feb	30	40–77	58.43 ± 9.35	50	100	Gillnet	Commercial
		37G1	54244	11.344	2016	Feb/Mar	19	34–72	51.85 ± 15.12	58	100	Bottom trawl	Survey
		37G1	54.11	11.213	2016	Jul	1	44	44 ± 0	0	100	Gillnet	Commercial
		38G0	54.438	10.689	2016	Mar	1	65	65 ± 0	0	100	Bottom trawl	Survey
		38G1	54.416	11.429	2016	Mar	4	26–68	41.75 ± 15.44	50	100	Bottom trawl	Survey
23	<20	40G2	55.945	12.712	2016	Mar	59	29–55	38 ± 4.75	32	24	Fishing rod	Recreational
24	40–45	37G3	54.278	13.892	2016	May	60	42–50	47.1 ± 2.06	32	0	Gillnet	Commercial
		37G3	54.28	13.892	2015	Oct	60	43–50	46 ± 1.95	38	0	Gillnet	Commercial
		37G4	54.409	14.11	2016	Jun	25	37–67	44.92 ± 6.42	40	8	Gillnet	Commercial
		38G2	54.623	12.868	2016	Apr	60	49–58	53.55 ± 2.65	25	5	Gillnet	Commercial
		38G3	54.77	13.258	2016	May	60	38–45	40.5 ± 1.78	27	17	Bottom trawl	Commercial
		38G3	54.75	13.25	2015	Dec	60	38–52	44.15 ± 3.93	53	0	Bottom trawl	Commercial
		38G4	54.663	14.463	2015	Sep	60	38–56	41.6 ± 2.55	25	0	Bottom trawl	Commercial
25	70–100	38G5	54.817	15.383	2016	Feb	13	30–41	34.77 ± 2.93	85	100	Gillnet	Commercial
		38G5	54.833	15.567	2016	Mar	10	36–46	39.5 ± 3.69	30	100	Gillnet	Commercial
		39G5	55.143	15.733	2016	May	10	34–49	40.3 ± 4.71	30	100	Gillnet	Commercial
		39G5	55.167	15.25	2016	Dec	9	28–44	33.3 ± 5.85	44	NA	Bottom trawl	Survey
		39G6	55.144	16.67	2016	Jun	20	24–54	40.55 ± 7.45	50	100	Bottom trawl	Survey
26	100	38G9	54.776	19.22	2015	Sep	10	23–36	32.2 ± 3.71	40	100	Bottom trawl	Survey
		39G8	55.213	18.517	2015	Sep	10	33–44	36.5 ± 3.64	0	NA	Bottom trawl	Survey
		39G8	55.087	18.336	2016	Dec	9	33–47	39.44 ± 3.98	44	0	Bottom trawl	Survey
		41G8	56.33	18.199	2016	Mar	1	38	38 ± 0	0	0	Gillnet	Commercial

### Data analysis

For each locus, observed heterozygosity, expected heterozygosity and *F*_IS_ were calculated with R v3.4.1 [27] and the package HIERFSTAT v0.04 [28]. Classical χ^2^-tests based on the expected genotype frequencies calculated from the allelic frequencies were performed to test deviations from Hardy-Weinberg equilibrium (HWE) based on 10,000 permutations with the package PEGAS v0.1.0 [29]. Tests were run per sampling location (SD) and pooled over all sampling locations. Fixation indices were calculated according to [30] and levels of significance were assessed by 10,000 bootstrap permutations implemented in the package MMOD v1.3.3 [31]. For loci within chromosomal rearrangements (see S5 Table), population-wise bootstrapping of individual genotypes was used to infer the probability of a significant over- or underrepresentation of the presumed collinear allele by resampling 10,000 replicates [32] and sequential Bonferroni correction was applied to correct for multiple tests [33].

Pairwise fixation indices (*F*_ST_) between six sampling locations were calculated for the minimum and the extended panel using the package HIERFSTAT v0.04 [28,34]. Confidence intervals were estimated based on 10,000 permutation replicates. For samples obtained within the same subdivision but from different years, we calculated *F*_ST_-values to test for signs of temporal genetic variation. Since we found no significant genetic differentiation for samples from SD 26 between years, they were pooled for further analyses.

Individual assignment of Baltic cod was performed on the minimum and the extended panel based on a Bayesian clustering algorithm implemented in STRUCTURE v2.3.4 [35]. The number of genetic clusters that best fit the data was estimated using the Evanno method implemented in STRUCTURE HARVESTER [36]. The most likely number of populations (*k)* was two populations, thus 10 replicates assuming *k* = 2 were run to assign individuals to one of the two populations without use of prior information as to their geographic origin. The admixture model was run to estimate individual admixture proportions, where the estimated proportion of an individual’s genotype (q) is allowed to be a mixture of both parental populations. Each run consisted of a burnin period of 10,000 followed by 100,000 Markov chain Monte Carlo (MCMC) steps. To average cluster memberships CLUMPP v1.1.1 was used applying the full-search algorithm [37].

We also used a second, computationally faster approach to assign individuals to their population of origin based on Principal Component Analysis (PCA) implemented in the EIGENSOFT v5 software (smartpca) [38]. Here, the genotypes were normalized so that each SNP had a mean genotype value of 0 and normalized variances. From these normalized data, the covariance matrix among individuals was approximated. Smartpca runs a PCA on this matrix and outputs principal components (eigenvectors) and eigenvalues. We applied the option “lsqproject” to improve handling of missing genotype data. The parameter “poplistname” was set to infer eigenvectors using only individuals from a subset of all sampled populations, and then project individuals to be assigned onto those eigenvectors. Spawning individuals from SD 22 and SD 25 (Table 1) were used as reference samples. Tracy-Widom statistics were used to test the correlation of eigenvectors with the “true” axes of variation [38].

## Results

To determine the usefulness of the two assays containing diagnostic and biologically informative SNPs to discriminate between Baltic cod populations, we genotyped individuals obtained from across the entire southern Baltic Sea. Population-wise analysis only revealed systematic deviation from HWE for samples from the mixing area SD 24 (S6 Table). At individual loci, the global maximum *F*_ST_-value was 0.36 for the minimum panel, and *F*_ST_-values for 18 out of 20 loci were significantly different from zero (Table 2). *F*_ST_-values of diagnostic SNPs ranged from 0.00 to 0.36. For the extended panel, the global maximum *F*_ST_-value was 0.44, and *F*_ST_-values for 33 out of 38 loci significantly differed from zero (Table 2). SNPs located in regions of large chromosomal rearrangements on linkage groups (LG) 2, 7 and 12 were less divergent. SNPs located within candidate genes putatively important for adaptation, showed high genetic differentiation with *F*_ST_-values ≥ 0.2 at five out of eleven loci. Samples were most differentiated at the hemoglobin locus (LG02_14566823_CAN, *F*_ST_ = 0.44, CI = 0.37–0.52), whereas aquaporin and zona pellucida were least differentiated among the six samples (aquaporin: LG08_10382534_CAN, *F*_ST_ = 0.03, CI = -0.01–0.07; zona pellucida: LG08_20478224_CAN, *F*_ST_ = 0.06, CI = 0.01–0.11).

**Table 2:** Summary statistics per locus. The second column shows SNPs belonging to the minimum (M) or extended (E) panel. Observed (*H*_obs_) and expected (*H*_exp_) heterozygosity are given for each locus of the extended panel. Global *F*_ST_-values and the confidence interval (CI) for each locus were calculated after [30]. For more information on annotated gene functions see S5 Table.

Locus	Panel	*H*_obs_	*H*_exp_	*F*_ST_	95% CI
*Diagnostic SNPs*					
LG01_10417249_SEL	M, E	0.291	0.498	0.220	0.138–0.302
LG02_01358822_SEL	M, E	0.297	0.500	0.253	0.168–0.338
LG02_14506653_SEL	M, E	0.395	0.500	0.171	0.097–0.244
LG03_07053408_SEL	M, E	0.229	0.473	0.146	0.080–0.212
LG04_23059256_SEL_60	M, E	0.374	0.495	0.252	0.166–0.337
LG06_00784232_SEL	M, E	0.449	0.492	0.174	0.101–0.245
LG07_23819790_SEL	M, E	0.320	0.269	0.230	0.175–0.284
LG09_08678526_SEL	M, E	0.240	0.475	0.292	0.215–0.370
LG09_16657913_SEL	M, E	0.352	0.388	0.005	-0.027–0.037
LG11_01922930_SEL	M, E	0.240	0.211	0.073	0.036–0.110
LG11_20277669_SEL	M, E	0.229	0.437	0.355	0.299–0.411
LG12_07553923_SEL	M, E	0.329	0.492	0.204	0.131–0.276
LG12_11560045_SEL	M, E	0.355	0.416	0.104	0.037–0.172
LG16_22359890_SEL	M, E	0.100	0.120	0.039	0.013–0.064
LG17_09361714_SEL	M, E	0.413	0.415	0.001	-0.039–0.040
LG18_04074216_SEL	M, E	0.444	0.469	0.258	0.201–0.316
LG18_17089172_SEL	M, E	0.217	0.304	0.144	0.091–0.197
LG21_04164158_SEL	M, E	0.475	0.494	0.117	0.050–0.184
LG21_18500787_SEL	M, E	0.416	0.495	0.137	0.061–0.212
LG22_04333346_SEL	M, E	0.393	0.448	0.116	0.052–0.179
*SNPs within candidate genes*					
LG01_14112750_CAN	E	0.313	0.498	0.228	0.160–0.296
LG02_01362812_CAN	E	0.214	0.450	0.342	0.279–0.404
LG02_01363907_CAN	E	0.285	0.500	0.307	0.229–0.385
LG02_14566823_CAN	E	0.303	0.500	0.442	0.366–0.518
LG02_14570979_CAN_3	E	0.239	0.393	0.218	0.152–0.284
LG07_03524202_CAN	E	0.410	0.499	0.092	0.047–0.138
LG08_10382534_CAN	E	0.222	0.274	0.031	-0.011–0.073
LG08_20478224_CAN	E	0.476	0.497	0.063	0.014–0.111
LG12_06163312_CAN	E	0.297	0.421	0.074	0.019–0.130
LG21_07486768_CAN	E	0.372	0.490	0.134	0.065–0.203
LG21_08595680_CAN	E	0.372	0.492	0.092	0.034–0.150
*SNPs within inversions*					
LG02_18724285_I02	E	0.135	0.469	0.283	0.217–0.348
LG02_20868512_I02	E	0.435	0.500	0.170	0.101–0.239
LG07_14812281_I07	E	0.312	0.430	0.112	0.048–0.177
LG07_16410308_I07	E	0.394	0.454	0.030	-0.012–0.071
LG07_20281433_I07b	E	0.327	0.489	0.095	0.035–0.154
LG12_11630885_I12	E	0.407	0.467	0.067	0.009–0.125
LG12_12529238_I12	E	0.396	0.471	0.046	-0.005–0.098

Inferred genetic relationships among samples were similar for the minimum and the extended panel with an identical overall level of differentiation (*F*_ST_ = 0.12 for both panels). The mean pairwise *F*_ST_-values based on the minimum and the extended panel were 0.39 and 0.35 (Tables 3 and 4) for reference cod collected in SD 22 (western) and SD 25 (eastern), respectively, with both values being significantly different from zero (CI = 0.28–0.41 and 0.31–0.46).

**Table 3:** Pairwise fixation indices (*F*_ST_) based on 20 diagnostic SNPs (minimum panel) to differentiate between western and eastern Baltic cod stock (N = 554). *F*_ST_-values are given below the diagonal, 95% confidence intervals are given above the diagonal. Significant values are marked in bold.

Subdivision	SD 22	SD 23	SD 24 (2015)	SD 24 (2016)	SD 25	SD 26
**SD 22**		0.01–0.07	0.11–0.18	0.14–0.23	0.31–0.46	0.31–0.46
**SD 23**	**0.03**		0.08–0.16	0.09–0.19	0.24–0.42	0.25–0.42
**SD 24 (2015)**	**0.15**	**0.12**		0.00–0.03	0.06–0.13	0.07–0.14
**SD 24 (2016)**	**0.19**	**0.14**	0.01		0.04–0.11	0.05–0.11
**SD 25**	**0.39**	**0.34**	**0.10**	**0.08**		-0.01–0.05
**SD 26**	**0.41**	**0.36**	**0.12**	**0.09**	0.02	

**Table 4:** Pairwise fixation indices (*F*_ST_) based on 38 SNPs (extended panel) to differentiate between western and eastern Baltic cod stock (N = 554). *F*_ST_-values are given below the diagonal, 95% confidence intervals are given above the diagonal. Significant values are marked in bold.

Subdivision	SD 22	SD 23	SD 24 (2015)	SD 24 (2016)	SD 25	SD 26
**SD 22**		0.03–0.15	0.10–0.15	0.13–0.20	0.28–0.40	0.28–0.40
**SD 23**	**0.09**		0.11–0.18	0.12–0.23	0.27–0.42	0.28–0.44
**SD 24 (2015)**	**0.13**	**0.15**		0.01–0.04	0.07–0.12	0.07–0.15
**SD 24 (2016)**	**0.17**	**0.18**	**0.02**		0.05–0.09	0.05–0.09
**SD 25**	**0.35**	**0.35**	**0.10**	**0.07**		0.00–0.03
**SD 26**	**0.36**	**0.37**	**0.12**	**0.08**	0.01	

**Fig 2:**
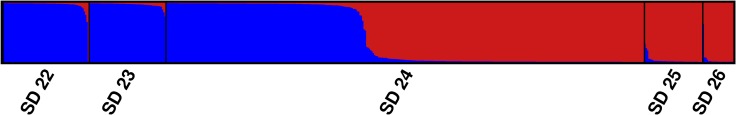
Assignment of Baltic cod (N = 554) inferred from model-based clustering with STRUCTURE at *k* = 2 using 20 SNP loci.

The Evanno method suggested *k* = 2 as the most likely number of clusters resulting from our data. Individual cod were assigned to the cluster in which the genotype of the individual was most likely to occur first with a Bayesian clustering approach. Samples collected in SD 22 and SD 25 were assigned to the western or eastern Baltic cod stocks with 98.5% and 100%, respectively, and the minimum and the extended panel yielded very similar results. Within SD 22 a single individual spawning in July was identified as an eastern Baltic cod. Specimens collected in SD 23 were all assigned to western Baltic cod. Specimens from the most eastern sampling location in SD 26 were classified as eastern Baltic cod at 97.6% confidence with the minimum panel and at 100% using the extended panel. Cod individuals from the mixing area Arkona (SD 24) were assigned to both stocks: 41.6% were assigned to the western Baltic cod stock, while 58.4% were assigned to the eastern Baltic cod stock. Individual assignments to the western or the eastern Baltic cod stock were identical, irrespective of which panel was used. In total, 554 out of 603 genotyped individuals (92%) could be assigned unambiguously to one of the two Baltic cod stocks (Figs 2 and S1).

**Fig 3:**
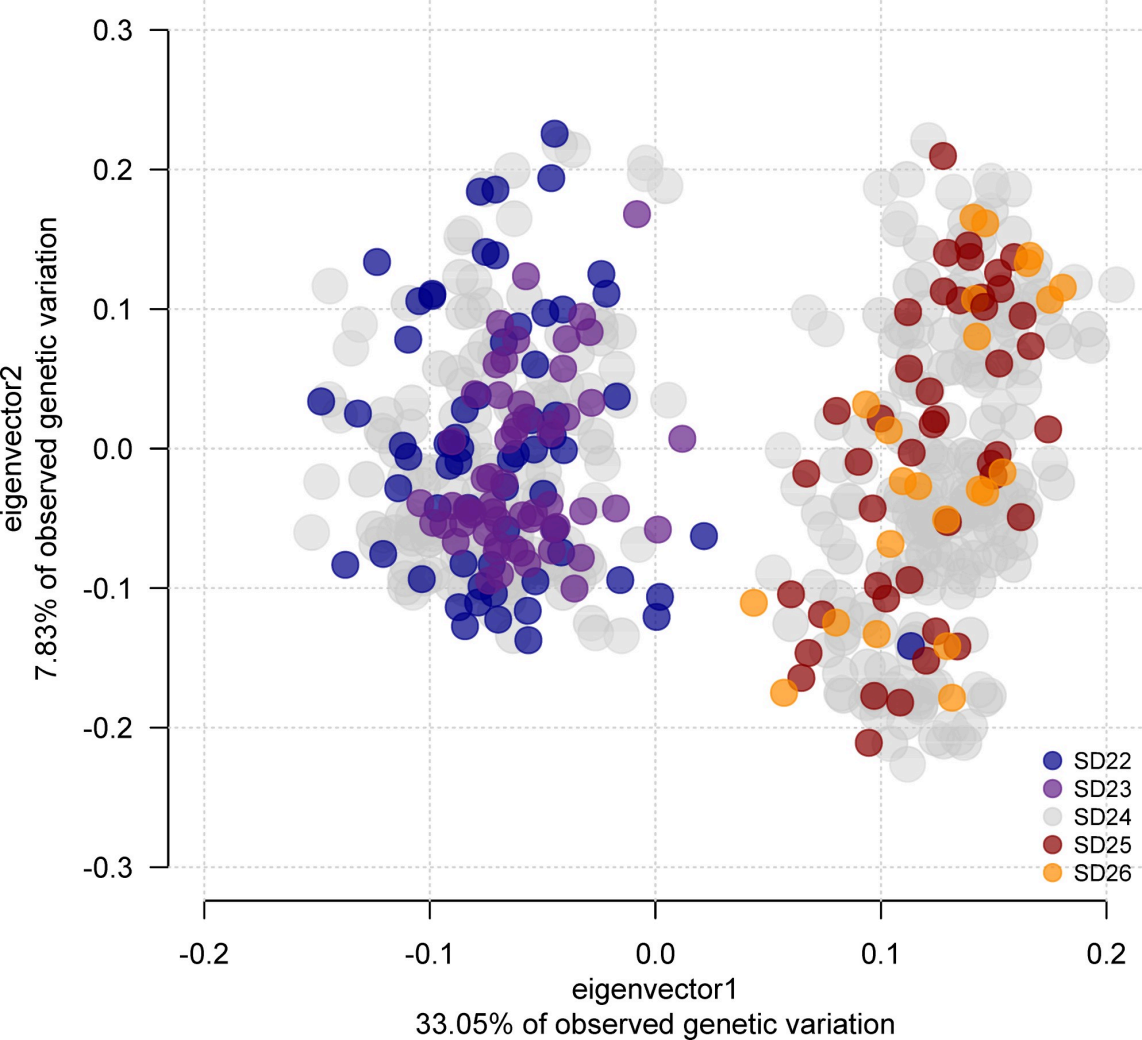
Principal component analysis of Baltic cod genotypes using the extended SNP panel comprising 38 SNPs. Eigenvectors were inferred with samples of spawning cod from ICES subdivisions (SD) 22 and 25. Points are color-coded according to locations within subdivisions. Total N = 554. Note the blue point (one eastern Baltic cod caught in SD 22) in the eastern Baltic cod cluster on the right.

A PCA showed two well separated clusters: the first cluster contained individuals from SD 22 and SD 23, the other cluster comprised individuals from SD 25 and SD 26 (Fig 3). For the extended panel, axes 1 and 2 explained 33.05% and 7.83% of the variation, respectively; for the minimum panel, axes 1 and 2 explained 38.12% and 7.08%, respectively, thus they represent the “true” axes of variation as endorsed by Tracy-Widom statistics (Table 5, [36]). Samples from SD 24 split between both clusters. PCA-based individual assignment of mixed stock samples to their respective population of origin was in complete concordance with results obtained using the Bayesian clustering approach implemented in STRUCTURE.

**Table 5:** Results shown for Tracy-Widom tests. Tracy-Widom tests and associated P-values for the significance of most informative eigenvectors (EV)) for the minimum and the extended SNP panel (N = 554).

	minimum		extended	
EV	Twstat	P	Twstat	P
1	2.389	< 0.01	5.388	< 0.01
2	-1.379	1	2.049	0.02
3	-0.697	1	3.494	< 0.01
4	-1.003	1	3.773	< 0.01

**Fig 4:**
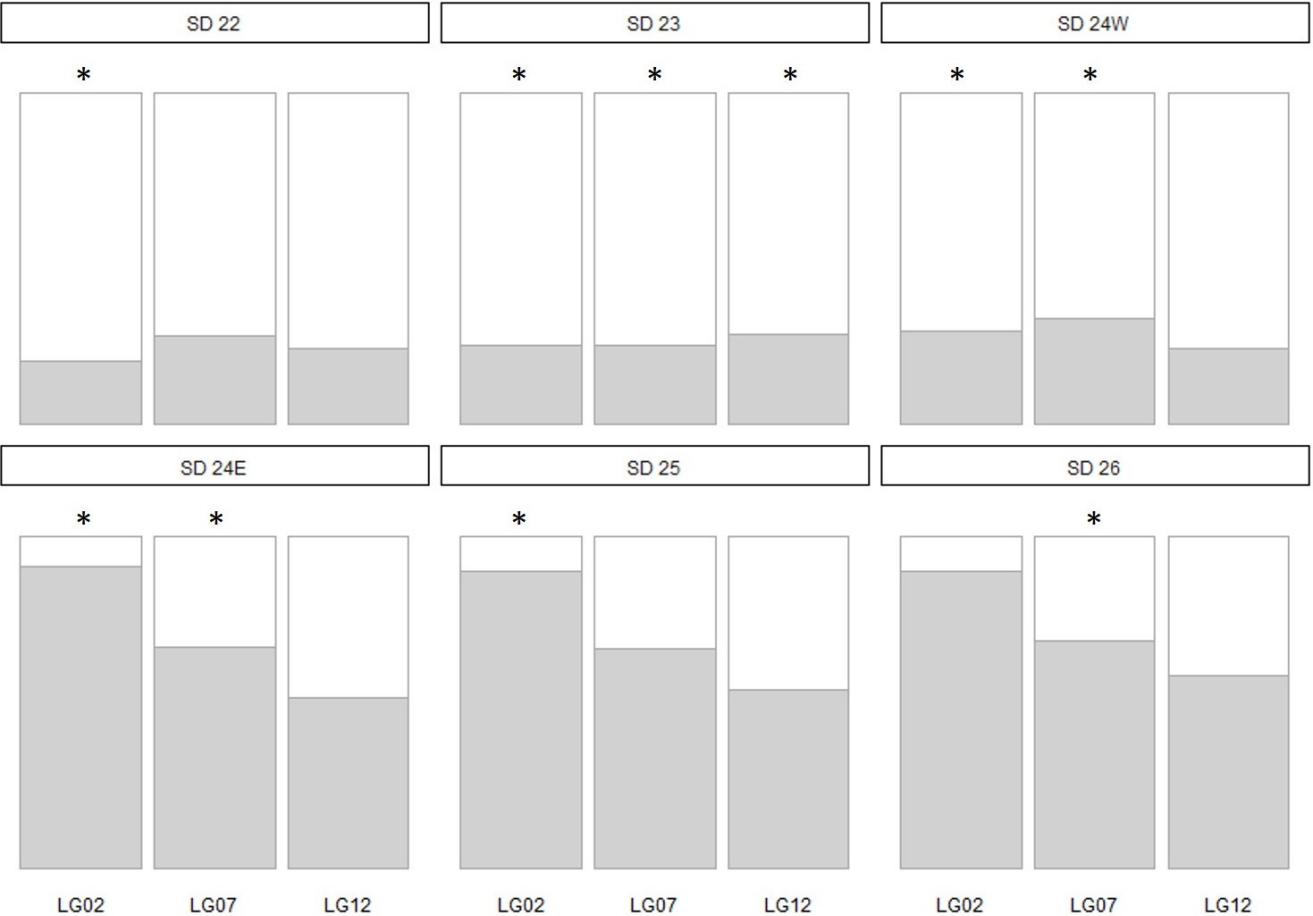
Allele frequency distribution of three inversions on LG 2, 7 and 12 in Baltic cod samples obtained from SD 22–26. Samples from the transition area in SD 24 were split in individuals genetically assigned to the western (SD 24W) and to the eastern (SD 24E) Baltic cod stock (see Figs 2 and 3). White: the proportion of the presumed collinear allele; grey: proportion of the presumed rearranged allele. Asterisks denote significant over- or underrepresentation of the presumed collinear allele.

Patterns of divergence at genomic loci within regions of chromosomal rearrangements were examined for cod in the Baltic Sea. We found a clear and statistically significant shift of allele frequencies within LG 2, 7 and 12 (Fig 4) corresponding to the geographic distribution of cod spawning communities, with the strongest signal for LG 2. Allele frequencies of cod from SD 23, SD 25 and SD 26 were not significantly different for SNPs on LG 7 and 12.Within the transition area (SD 24), the allele frequency distributions for individuals assigned to the western or eastern Baltic cod stock were congruent with the allele frequency patterns found in the respective parent population.

## Discussion

Unravelling the spatial and temporal distribution of fish stocks is essential for a proper assessment and management of different stock components [1]. Here, we used molecular markers to assign individuals to their respective population of origin to provide a tool for exploring variation in Baltic cod stock contributions and catch compositions. Based on whole-genome re-sequencing information from reference samples caught in the central Baltic and the transition area to the North Sea, loci with high discriminatory power between the western and eastern Baltic cod stocks were identified and validated. Furthermore, the application of the extended panel revealed remarkable differences between the two populations in the allele frequency distributions at loci potentially important for local adaptation to the environment of the Baltic Sea.

### Establishment of new SNP panels and population structure of cod in the Baltic

We observed strong genetic differentiation between western and eastern locations, confirming the general validity of stock designations. At the same time we also confirm considerable mixing of the two populations in SD 24.

So far, methods for discriminating western and eastern Baltic cod were mainly based on phenotypic differences [39] and molecular techniques [17,18,40,41] with variable levels of accuracy. More recently, specimens were genetically characterized as originating from either of the two Baltic cod stocks by utilizing a high-graded 39 SNP-array [14,19]. In contrast to previous approaches for generating diagnostic marker panels for Baltic cod populations [7,14,19], we have included whole-genome information on genetic variation between cod populations sampled in the study area. We used more than 68,000 SNPs (see S1 File) to harvest our SNPs from, thus better representing the entire genome, compared to approximately 1,200 SNPs used for generating the existing high-graded 39 SNP-array. Consequently, the resolving power of our diagnostic marker set is increased, while at the same time the number of markers needed is reduced. Furthermore, by referring to a high-quality reference genome for Atlantic cod [20] we were able to select our SNP loci based on their genomic position, avoiding the integration of physically linked SNPs. Hence, the new minimum panel for discriminating between Baltic cod populations is a major improvement over existing SNP panels, because it accounts for the species’ specific genome architecture [21–23,25]. Diagnostic markers from highly linked genomic regions could thus be excluded from the panel *a priori*, to avoid potential biases in population assignment. The minimum panel introduced in the current study allows a more cost-efficient throughput of a large number of samples by minimizing the need for consumables and reagents, while maintaining a high level of confidence at identifying the individual’s source population. Moreover, our minimum panel can easily be extended by additional loci (this study, [7,42]) to gain extra biological information or to modify the spatial resolution of population structure, thereby providing the flexibility to address a wide suite of biological and management questions.

### Applications to questions of stock discrimination and mixing of Baltic cod populations in SD 24

The use of our markers confirmed the co-occurrence of both western and eastern Baltic cod stocks in the Arkona Sea [14,19], but mixing may not be restricted to this area.

Results of a multi-year otolith analysis of stock mixing suggested that eastern Baltic cod have been abundant in the Arkona Basin during the last two decades with a substantial increase from 30% to around 70% in the late 2000s [14]. Nevertheless, there are still uncertainties whether the increase of eastern Baltic cod abundance in the Arkona Basin is an autonomous production from SD 24 or related to a spill-over of cod originating from SD 25 or to shifts in environmental conditions [14,43]. There is a clear need to assign individuals correctly to their native stock, so as to allow the monitoring of both populations as well as the identification of stock-specific or trait-specific (e.g. length, sex, age) movement patterns within the southern Baltic, and to resolve catch compositions from the different nations fishing in the Arkona Sea. The molecular approach presented here has proven useful for differentiating between individual western and eastern Baltic cod.

We recorded one spawning female eastern Baltic cod in the western Baltic (SD 22), which is consistent with observations that have been made also in previous studies [19,44]. Such occurrences suggest that mixing might also affect areas beyond the Arkona Basin. Hence, future work on mixing dynamics of cod in the southern Baltic should also consider adjacent areas to improve our understanding of cod migrations.

A moderate number of individuals could not unequivocally be determined as originating from the western or the eastern Baltic cod population. Methodological inaccuracies cannot be fully avoided due to factors hampering the determination of the different source populations. In our case, most of these samples that failed an assignment were characterized by high levels of missing data across all loci. The non-assigned samples originated from the total study area with a slight overrepresentation of the ones caught in SD 24 (26 out of 49). But compared to the total number of sampled individuals per ICES subdivision, only 7% of all samples caught in SD 24 failed an assignment, but 24% of all samples from SD 25. However, some of the individuals that failed to be assigned might be hybrids of mixed ancestry. There is an ongoing debate on the possibility of hybridization between western and eastern Baltic cod [17,19,40], but it is presently assumed that differences in the timing of spawning seasons and geographically segregated spawning grounds with different environmental conditions (i.e. with eastern Baltic cod spawning later in the season, further east, in deeper waters) provide effective barriers restricting gene flow between the two Baltic cod populations and stimulating reproductive isolation [9]. Interestingly, hybridization between the western and the eastern Baltic cod stock seems to be rare in the mixing zone as suggested by the low individual admixture proportions in our analyses and previous findings support a scenario of mechanical mixing as being the predominant form of interaction between the two stocks and hybridization as being only occasional [19]. The reproductive activity of western and eastern Baltic cod spatially and temporarily overlaps at least to some extent, which raises the question about mechanisms of reproductive isolation preventing natural hybridization.

### Applications to questions of local adaptation of cod to the Baltic environment

In the Baltic Sea, the steep gradient from high salinity waters in the Kattegat to regions of lower salinity in the eastern- and northernmost parts limits the spatial distribution of marine organisms, and in the case of the Baltic cod, successful reproduction [45–48]. The Baltic Sea therefore constitutes an ideal model system to assess questions pertaining to local adaptations along environmental gradients [49]. Previous results from high density SNP-arrays indicated that in Baltic cod loci under directional selection were strongly correlated with habitat differences in salinity, oxygen and temperature, and that a substantial amount of divergence was driven by adaptation to low salinity [9]. By focusing on genes related to environmental conditions that are characteristic for a life in the eastern Baltic Sea, we found the most pronounced genetic differences at the hemoglobin and the prolactin loci. The hemoglobin polymorphism in Atlantic cod has been extensively assessed in the past [8,40]. The polygenic nature of hemoglobin enables cod to cope with chronic hypoxia or long-term changes in temperature by altering gene expression levels between hemoglobin isoforms [8]. Western Baltic cod live in the western, shallower areas with higher salinities; thermal convection results in full vertical mixing each winter and low oxygen conditions only occur in summer and autumn. In contrast, bathymetry and stratified hydrography in the deeper basins of the eastern Baltic Sea regularly lead to challenging conditions for cod. As a result, eastern Baltic cod live at the edge of the species‘ physiological limits and have to cope with low-oxygen conditions by expressing a hemoglobin variant with high oxygen-affinity [50–53]. Similarly important, prolactin is essential for the acclimatization to a hypo-osmotic medium by changing cellular water and ion permeability [54]. The variation in allele frequencies found between both populations thus may represent adaptive genotypic responses to habitat differences of the two Baltic cod populations.

In Atlantic cod, four large chromosomal inversions (e.g. on LG 1, 2, 7 and 12) have been identified to discriminate between cod populations throughout its geographical distribution, i.e. dominating the observed genomic divergence by large allele frequency shifts [22,23,55], whereas the rest of the genome displays low levels of genomic differentiation. These patterns strongly suggest that these inversions are maintained by selection, and thus play a major role in local adaptation to environmental conditions linked to oxygen and salinity [9] as well as migratory behaviour [21,23,56]. The occurrence of three distinct chromosomal inversions have recently been shown to provide a genomic basis for fine-scale local adaptation in spite of the species’ general potential for panmixia in its southern distribution [25,57]. For our extended panel, we chose SNPs from each of the three linkage groups (LG 2, 7 and 12) to investigate the distribution of alleles within the Baltic Sea. We found that allele frequencies of the collinear and the inverted allele mirror the population structure results of Baltic cod. However, for LG 7 we observed a more pronounced signal of divergence compared to LG 2 and LG 12, which might be due to reduced recombination or selection pressure.

The application of the new panels introduced here in genetic monitoring and in studies of the genetic signatures of local adaptation, holds strong potential to improve the sustainable management of western and eastern Baltic cod populations and to enhance our understanding of the evolutionary importance of genes related to local adaptation.

## Supporting information

S1 File Supplementary material and results. Supplementary information on whole-genome re-sequencing reference Atlantic cod samples used for variant identification. Details on variant calling and design of the minimum and extended SNP-panels are given.
(DOCX)

S1 Table Details on specimen used for whole-genome sequencing. (XLSX)

S2 Table Pairwise fixation index (*F*_ST_) values (Weir & Cockerham 1984) for each locus. SNPs selected for differentiating between KBI and BOR are commented.
(XLSX)

S3 Table Probability of correct reassignment of specimens ARK and ORE to the reference populations NOR, KBI, and BOR for five replicates. (XLSX)

S4 Table Pairwise *F*_ST_ estimates (Weir & Cockerham, 1984) and p-values for samples used for whole-genome re-sequencing. (XLSX)

S5 Table SNP markers, positions, gene location, and flanking regions of the minimum and the extended SNP-panel. (XLSX)

S6 Table Observed (*H*_obs_) and expected (*H*_exp_) heterozygosity per ICES subdivision (SD) 22–26. Loci significantly deviating from Hardy-Weinberg-Equilibrium are indicated in bold.
(XLSX)

S1 Fig Assignment of Baltic cod (N = 554) inferred from model-based clustering with STRUCTURE at *k* = 2 using 38 SNP loci. (TIF)
