# Supplementary material for: Assessing SNP-markers to study population mixing and ecological adaptation in Baltic cod
Source: PLoS One. 2019 Jun 20;14(6):e0218127. doi: 10.1371/journal.pone.0218127 (PMC6586271; doi:10.1371/journal.pone.0218127)
Supplement: S1 File — Supplementary information on whole-genome re-sequencing reference Atlantic cod samples used for variant identification. Details on variant calling and design of the minimum and extended SNP-panels are given. (DOCX) [file pone.0218127.s001.docx]

**Assessing SNP-markers to study population mixing and ecological adaptation in Baltic cod**

Peggy Weist^1*^, Franziska M Schade^2^, Malte Damerau^1^, Julia MI Barth^3,4^, Jan Dierking ^5^, Carl André^6^, Christoph Petereit^5,#^, Thorsten Reusch^5^, Sissel Jentoft^4^, Reinhold Hanel^1^, Uwe Krumme^2^

^1^Thünen-Institute of Fisheries Ecology, Bremerhaven, Germany

^2^Thünen-Institute of Baltic Sea Fisheries, Rostock, Germany

^3^Zoological Institute, University of Basel, Basel, Switzerland

^4^Centre for Ecological and Evolutionary Synthesis, Department of Biosciences, University of Oslo, Oslo, Norway

^5^GEOMAR Helmholtz Centre for Ocean Research, Kiel, Germany

^6^Department of Marine Sciences-Tjärnö, University of Gothenburg, Strömstad, Sweden

^#^Current address: Bruno-Lorenzen-Schule, Schleswig, Germany

^*^Corresponding author:

Email: [peggy.weist@thuenen.de](mailto:peggy.weist@thuenen.de)

# Supplementary Methods

**Whole genome-sequencing and SNP identification**

For the selection of diagnostic SNPs, we mined whole genome-sequencing data from a total of 115 cod specimens (S1 Table) originating from the North Sea (NOR, SD 4.b), western Baltic Sea (Öresund (ORE, SD 23), Kiel Bight (KBI, SD 22), Arkona Sea (ARK, SD 24)), and eastern Baltic Sea (Bornholm Basin (BOR, SD 25)). Briefly, DNA was extracted from muscle tissue stored in pure ethanol or from dried fin clips stored in paper bags or in pure ethanol, respectively, using the DNeasy Blood & Tissue kit (Qiagen, Venlo, Neatherlands). Library preparation and sequencing was performed at the Norwegian Sequencing Centre on an Illumina HiSeq 2500 (125 bp paired-end) to about ~9x coverage. Reads were mapped using the algorithm BWA-MEM in bwa v0.7.5 [1] against the Atlantic cod gadMor2 genome assembly [2]. Variant calling was performed using the gatk GENOTYPECALLER v3.3.0 [3] and filtered according to gatk’s Best Practices recommendations: FS > 60.0, MQRankSum < -12.5, ReadPosRankSum < -8.0, QD < 2.0, MQ < 40.0 [4]. After removal of multi-allelic SNPs and indels, all sites with a genotype quality score < 20, and read depth (DP) < 3, or DP > 20 were replaced with missing data for the respective individual. SNPs with a minor-allele frequency ≤ 3%, missing data > 10% and heterozygous excess (*p*< 0.001) were removed using plink v1.90 beta [5], resulting in a final dataset of 68 753 SNPs.

To estimate population divergence, pairwise fixation indices (*F*_ST_, [8]) between samples were calculated using arlequin v3.5 [6] and arlecoremac_64bit v3.5.2.2 [6]. Significance was assessed using 10 000 permutation steps (S2 Table) and *p*-values were adjusted for multiple testing by applying a false discovery rate (FDR) approach for non-independent tests [7]. For the selection of SNPs with high discriminatory power between populations, *F*_ST_ values [8] for each SNP locus were calculated between the samples KBI-BOR, KBI-NOR, KBI-NOR/BOR, and NOR-BOR by applying the “--weir-fst-pop” flag in vcftools v0.1.14 [9] (S2 Table). Candidate SNPs for the minimal marker panel were selected based on their *F*_ST_ values for each pairwise analysis, and the most differentiated loci per comparison were selected as diagnostic SNPs. To avoid linkage-disequilibrium from physically linked SNPs, markers were further selected to be from different linkage groups when possible, and SNPs located on the same linkage groups had a minimum distance of 4 Mb. Some high *F*_ST_ SNPs were dropped from the panel since their flanking regions were not suitable for primer design. The assignment power of 23 selected SNPs (S2 Table) was tested using Geneclass v2 [10–12] using the samples ARK and ORE as populations to be assigned, and BOR, KBI, and NOR as reference populations. All assignment tests were replicated five times and probability calculations were generated using 10,000 simulated individuals with an FDR < 0.01 (S3 Table). Individuals were considered to be correctly assigned if the inferred probability for assignment to the true population of origin was higher than 70% (see [1] for concept). We defined the “true population of origin” for the individuals to be assigned based on pairwise *F*_ST_ values (S4 Table): since KBI/ORE and BOR/ARK were not genetically different, we considered the “true” population for ORE to be KBI and for ARK to be BOR, respectively. Thus, ORE and ARK were used as holdout datasets in the assignment tests.

In addition to the population-diagnostic SNPs, three markers suitable for sex-determination were selected based on the study from Star et al. [14], and nine markers suitable to distinguish between inverted and collinear regions on linkage group (LG) 2, 7 and 12 were selected from the regions previously described for Atlantic cod [15–17] (S5 Table), likely harboring loci important for local adaptation [18]. Furthermore, we added thirteen SNPs associated with life-history trait candidate genes potentially important for adaptation to changing environmental conditions in the Baltic Sea, such as low salinity conditions, thermal stress and oxygen deficiency (S5 Table). To assess the assignment power of the 48 SNPs selected in total, we again conducted assignment-tests using the same procedure as described above.

# Supplementary results and discussion

Based on whole genome re-sequencing efforts, the analysis of five Atlantic cod population samples resulted in a total of 68.8K SNPs, which were used to select a minimum set of diagnostic markers suitable for the assignment of Atlantic cod to the western or eastern Baltic stock. For the reference samples only used for the selection of SNPs, pairwise *F*_ST_ values between KBI and ORE, as well between BOR and ARK were low and non-significant (S4 Table). Therefore, only specimens from KBI, BOR and NOR were used to select the diagnostic SNPs, while specimens from ORE and ARK were used to test their power of assignment. Using the minimal panel of 23 diagnostic markers, 100 ± 0% ARK specimens were unambiguously assigned to BOR, and 95.2 ± 0% ORE specimen were assigned to KBI; 4.8% were misassigned to NOR (S3 Table). By using the full panel of 48 markers, 100 ± 0% ARK specimen were assigned to BOR, and 88.9 ± 0% ORE specimen were assigned to KBI. Again, the 11.1% of the individuals were misassigned to NOR.

Comparing the sequence information of SNPs presented in this study with previously published SNPs to distinguish between western and eastern Baltic cod stocks [19] revealed, that all SNPs from the minimum panel were original. However, LG02_14570979_CAN_3 from the full panel corresponds to Gm_HbBeta1_1 used by Hemmer-Hansen et al. [19].

Interestingly, we found no mixture of both Baltic cod populations in the ARK sample caught in 2012, but 100% of the individuals were assigned to the eastern Baltic population which is contrary to our results based on genotyping individuals from SD 24 caught in 2015 and 2016. Interannual fluctuation in the proportion of mixing of western and eastern Baltic cod is expected to a certain degree [19,20]. However, the contrasting findings might be attributed to the different sampling strategies intended for the baseline samples, that were sequenced (one sampling site in May in eastern part of SD 24), and the samples, that were genotyped for evaluating the performance of the selected markers (year-round samples from different sampling sites, see Table 1).

# Supplementary literature cited

1. Li H, Durbin R. Fast and accurate short read alignment with Burrows–Wheeler transform. Bioinformatics. 2009;25: 1754–1760. doi:10.1093/bioinformatics/btp324

2. Tørresen OK, Star B, Jentoft S, Reinar WB, Grove H, Miller JR, et al. An improved genome assembly uncovers prolific tandem repeats in Atlantic cod. BMC Genomics. 2017;18: 060921. doi:10.1186/s12864-016-3448-x

3. DePristo M a., Banks E, Poplin RE, Garimella K V., Maguire JR, Hartl C, et al. A framework for variation discovery and genotyping using next- generation DNA sequencing data. Nat Genet. 2011;43: 491–498. doi:10.1038/ng.806.A

4. Van der Auwera GA, Carneiro MO, Hartl C, Poplin R, del Angel G, Levy-Moonshine A, et al. From fastQ data to high-confidence variant calls: The genome analysis toolkit best practices pipeline. Curr Protoc Bioinforma. 2013;11: 11.10.1-11.10.33. doi:10.1002/0471250953.bi1110s43

5. Purcell S, Neale B, Todd-Brown K, Thomas L, Ferreira MAR, Bender D, et al. PLINK: A tool set for whole-genome association and population-based linkage analyses. Am J Hum Genet. 2007;81: 559–575. doi:10.1086/519795

6. Excoffier L, Lischer HEL. Arlequin suite ver 3.5: A new series of programs to perform population genetics analyses under Linux and Windows. Mol Ecol Resour. 2010;10: 564–567. doi:10.1111/j.1755-0998.2010.02847.x

7. Benjamini Y, Yekutieli D. The control of the false discovery rate in multiple testing under dependency. Ann Stat. 2001;29: 1165–1188. doi:10.1214/aos/1013699998

8. Weir BS, Cockerham CC. Estimating F-Statistics for the analysis of population structure. Evolution (N Y). 1984;38: 1358. doi:10.2307/2408641

9. Danecek P, Auton A, Abecasis G, Albers CA, Banks E, DePristo MA, et al. The variant call format and VCFtools. Bioinformatics. 2011;27: 2156–2158. doi:10.1093/bioinformatics/btr330

10. Rannala B, Mountain JL. Detecting immigration by using multilocus genotypes. Proc Natl Acad Sci. 1997;94: 9197–9201. doi:10.1073/pnas.94.17.9197

11. Paetkau D, Slade R, Burden M, Estoup A. Genetic assignment methods for the direct, real-time estimation of migration rate: A simulation-based exploration of accuracy and power. Mol Ecol. 2004;13: 55–65. doi:10.1046/j.1365-294X.2004.02008.x

12. Piry S, Alapetite A, Cornuet J-M, Paetkau D, Baudouin L, Estoup A. GENECLASS2: A software for genetic assignment and first-generation migrant detection. J Hered. 2004;95: 536–539. doi:10.1093/jhered/esh074

13. Leslie S, Winney B, Hellenthal G, Davison D, Boumertit A, Day T, et al. The fine-scale genetic structure of the British population. Nature. 2015;519: 309–314. doi:10.1038/nature14230

14. Star B, Tørresen OK, Nederbragt AJ, Jakobsen KS, Pampoulie C, Jentoft S. Genomic characterization of the Atlantic cod sex-locus. Sci Rep. 2016;6: 31235. doi:10.1038/srep31235

15. Kirubakaran TG, Grove H, Kent MP, Sandve SR, Baranski M, Nome T, et al. Two adjacent inversions maintain genomic differentiation between migratory and stationary ecotypes of Atlantic cod. Mol Ecol. 2016;25: 2130–2143. doi:10.1111/mec.13592

16. Sodeland M, Jorde PE, Lien S, Jentoft S, Berg PR, Grove H, et al. “Islands of divergence” in the Atlantic cod genome represent polymorphic chromosomal rearrangements. Genome Biol Evol. 2016;8: 1012–1022. doi:10.1093/gbe/evw057

17. Berg PR, Star B, Pampoulie C, Sodeland M, Barth JMI, Knutsen H, et al. Three chromosomal rearrangements promote genomic divergence between migratory and stationary ecotypes of Atlantic cod. Sci Rep. 2016;6: 23246. doi:10.1038/srep23246

18. Barth JMI, Berg PR, Jonsson PR, Bonanomi S, Corell H, Hemmer-Hansen J, et al. Genome architecture enables local adaptation of Atlantic cod despite high connectivity. Mol Ecol. 2017;26: 4452–4466. doi:10.1111/mec.14207

19. Hemmer-Hansen J, Hüssy K, Baktoft H, Huwer B, Bekkevold D, Haslob H, et al. Genetic analyses reveal complex dynamics within a marine fish management area. Evol Appl. 2019;00: 1–15. doi:10.1111/eva.12760

20. Hüssy K, Hinrichsen HH, Eero M, Mosegaard H, Hemmer-Hansen J, Lehmann A, et al. Spatio-temporal trends in stock mixing of eastern and western Baltic cod in the Arkona Basin and the implications for recruitment. ICES J Mar Sci. 2016;73: 293–303. doi:10.1093/icesjms/fsv227
