# Supplementary figures and images for: Assessing SNP-markers to study population mixing and ecological adaptation in Baltic cod
Source: PLoS One. 2019 Jun 20;14(6):e0218127. doi: 10.1371/journal.pone.0218127 (PMC6586271; doi:10.1371/journal.pone.0218127)

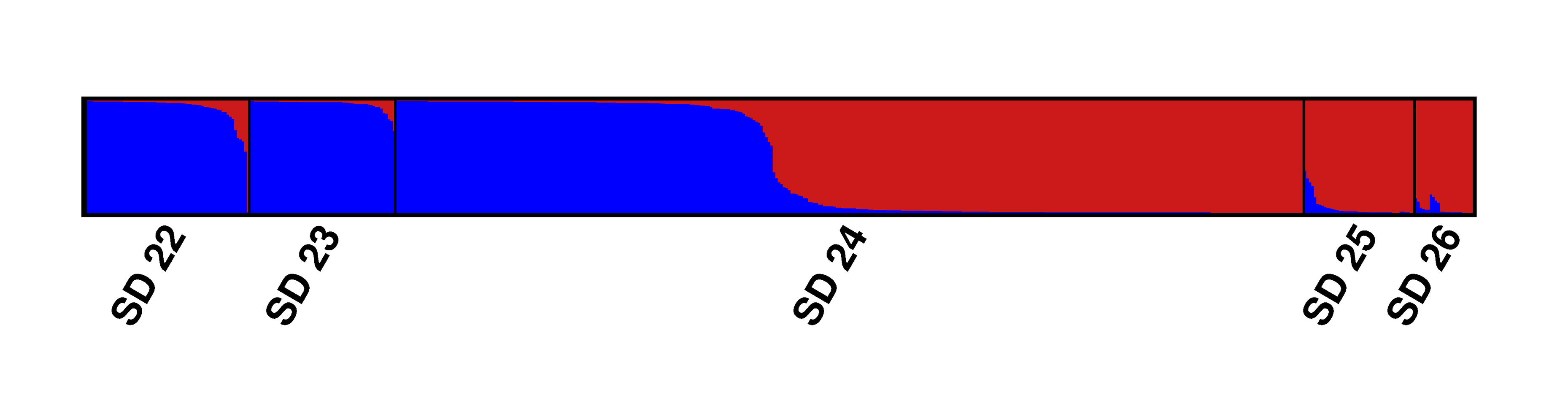

Supplement: S1 Fig — (TIF) [file pone.0218127.s008.tif]
